# Cholinergic Enhancement of Visual Attention and Neural Oscillations in the Human Brain

**DOI:** 10.1016/j.cub.2012.01.022

**Published:** 2012-03-06

**Authors:** Markus Bauer, Christian Kluge, Dominik Bach, David Bradbury, Hans Jochen Heinze, Raymond J. Dolan, Jon Driver

**Affiliations:** 1 Wellcome Trust Centre for Neuroimaging, University College London, London WC1N3BG, UK; 2 UCL Institute of Cognitive Neuroscience, University College London, London WC1N3AR, UK; 3 Otto-von-Guericke-Universität Magdeburg, 39120 Magdeburg, Germany

## Abstract

Cognitive processes such as visual perception and selective attention induce specific patterns of brain oscillations [1–6]. The neurochemical bases of these spectral changes in neural activity are largely unknown, but neuromodulators are thought to regulate processing [7–9]. The cholinergic system is linked to attentional function in vivo [10–13], whereas separate in vitro studies show that cholinergic agonists induce high-frequency oscillations in slice preparations [14–16]. This has led to theoretical proposals [17–19] that cholinergic enhancement of visual attention might operate via gamma oscillations in visual cortex, although low-frequency alpha/beta modulation may also play a key role. Here we used MEG to record cortical oscillations in the context of administration of a cholinergic agonist (physostigmine) during a spatial visual attention task in humans. This cholinergic agonist enhanced spatial attention effects on low-frequency alpha/beta oscillations in visual cortex, an effect correlating with a drug-induced speeding of performance. By contrast, the cholinergic agonist did not alter high-frequency gamma oscillations in visual cortex. Thus, our findings show that cholinergic neuromodulation enhances attentional selection via an impact on oscillatory synchrony in visual cortex, for low rather than high frequencies. We discuss this dissociation between high- and low-frequency oscillations in relation to proposals that lower-frequency oscillations are generated by feedback pathways within visual cortex [20, 21].

## Highlights

► Cholinergic agonist enhances human performance in a visuospatial attention task ► Occipital alpha/beta but not gamma oscillations under cholinergic control ► Alpha/beta attentional enhancement by cholinergic agonist relates to behavior ► Dichotomy of anatomical feedback versus feedforward connections may explain results

## Results

Neural processing of sensory signals originating from an attended location is thought to be enhanced by changes in oscillatory neural activity. Low-frequency alpha and beta oscillations in attended neuronal representations can be suppressed even before an expected stimulus appears (and enhanced for unattended) [5, 6]. This is thought to reflect up- and downregulation in the excitability of relevant neuronal populations [22]. Conversely, stimulus induced high-frequency gamma oscillations for attended neuronal representation are enhanced [1–4] and this is thought to increase their efficacy in driving postsynaptic neurons engendering privileged access to further processing stages [1, 23]. As for oscillations in general, the neurochemical pathways supporting these spectral changes are unknown but theoretical proposals suggest that an enhancement in high-frequency gamma oscillations is driven by cholinergic activity [17–19]. However, alpha oscillations are also known to be influenced by cholinergic neuromodulation [24–27].

**Figure 1:**
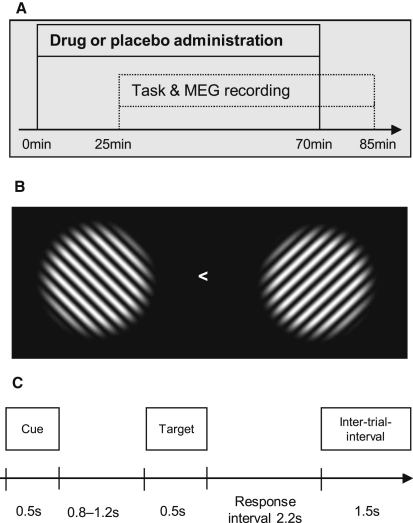
Experimental Timeline and Stimuli (A) Physostigmine or placebo was administered intravenously starting 25 min prior to onset of the visuospatial attention task and concurrent MEG recording, then continuing until 15 min prior to end of experimental session. (B) Each trial began with onset of a symbolic cue (right or left arrow, as shown) for 500 ms, indicating which hemifeld to attend. Participants fixated the central cross throughout the remainder of the trial, which comprised a 0.8–1.2 s (rectangular distribution) cue-target interval, followed by presentation of bilateral gratings for 500 ms, with up to 2.2 s for participants to make the tilt judgement (clockwise or counterclockwise relative to diagonal) for the grating in the attended hemifield. (C) Example display of bilateral gratings, spatial frequency 1.2 cycles/degree, circular window of 7 degrees, centered at 8 degrees eccentricity along the horizontal meridian.

Here we tested the impact of a cholinergic pharmacological intervention on brain oscillations during an attentional task in humans. Specifically, we recorded magnetoencephalography (MEG) while participants performed a spatial visual attention task (Figure 1), either under treatment with physostigmine [10, 11] as a cholinergic agonist or under placebo.

We recruited 16 participants who underwent both drug and placebo sessions (counterbalanced order) during this task. A central precue at trial start indicated which hemifield should be attended for a subsequently presented bilateral pair of gratings (see Figures 1B and 1C). The task was to discriminate orientation (clockwise or anticlockwise tilt relative to diagonal, titrated to yield ∼90% accuracy) for the attended hemifield on each trial.

Under physostigmine, performance was faster than placebo (mean 779.8 ms versus 819.2 ms, mean speeding of 39.4 ms) without accuracy cost (mean 90% correct under physostigmine, 89% for placebo, n.s.). This difference was significant for reaction time (RT, t = −1.84, p < 0.05), when the order of drug and placebo was taken into account, as well as for inverse efficiency (combining RT and accuracy into a single value [28], t = −2.52, p < 0.05), and for the latter this was significant also without taking the order-effect into account (t = −1.97, p < 0.05). Thus, the drug improved performance, extending previous demonstrations that cholinergic enhancement can improve attentional processing.

We performed a time-frequency (t-f) analysis on MEG time courses projected onto the cortical surface, using a source-reconstruction method (see Supplemental Experimental Procedures) similar to previous studies [3, 4] to test the impact of the cholinergic agonist on well-known changes in oscillatory activity related to visuospatial attention. Directing attention to the left or right hemifield is known to suppress contralateral and/or increase ipsilateral alpha/beta activity [5, 6, 22], whereas gamma synchronization is enhanced [1–4] contralateral to the attended hemifield in visual cortex. Accordingly we tested for the expected symmetric attentional “hemispheric lateralization” effects in visual cortex (see Experimental Procedures for our formal symmetry constraint), then assessed any impact of physostigmine versus placebo upon either alpha/beta or gamma spatial attention effects.

### Cholinergic Enhancement of Alpha/Beta Spatial Attention Effects

**Figure 2:**
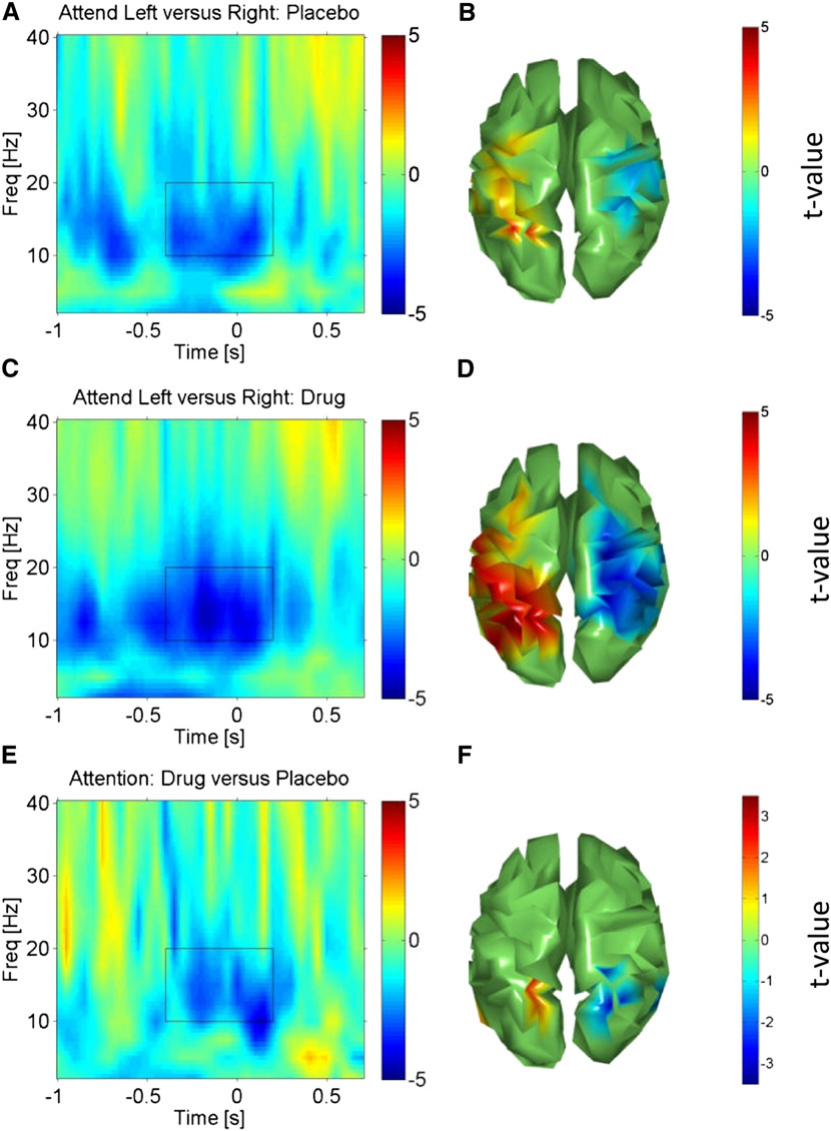
Spatial Attention and Alpha/Beta Oscillations (A) Time-frequency (t-f) profile for effect of spatial attention in the placebo session for symmetric hemispheric lateralization effects of Attention Left minus Attention Right at low frequency oscillations. Time zero corresponds to target onset in this and all subsequent t-f plots, and the color bar indicates t values. The t-f plot combines analogous effects in the left and right hemisphere. (B) The topography reveals suppressed/enhanced alpha/beta power (t-f window marked in A) in the hemisphere contralateral/ipsilateral to the attended hemifield, as expected (blue colors represent suppression, red enhancement). (C and D) T-f profile for corresponding effect of spatial attention in the physostigmine condition, with topography shown in (D); note the enhanced effect compared with (A) and (B). (E) T-f profile for the direct contrast of spatial attention effect in physostigmine minus placebo conditions, with topography shown in (F). (F) The cholinergic enhancement is localized to parieto-occipital cortex, an area tightly linked to alpha oscillations (see also Figure S1 for closer investigation of the parieto-occipital sulcus). Topographies are thresholded at p < 0.05, uncorrected, but for symmetric voxel pairs (see Experimental Procedures).

As expected [5, 6], alpha/beta hemispheric lateralization effects resulting from attended hemifield emerged in the preparatory cue period for occipital, parietal, and motor cortex (Figures 2A–2D), peaked around expected target onset, and then returned back to baseline levels. The novel result is that here alpha/beta spatial attention effects on visual cortex were enhanced by our cholinergic manipulation, being more pronounced under physostigmine than placebo (see Figures 2E and 2F for direct comparison, p < 0.0001, uncorrected). This cholinergic enhancement of alpha/beta spatial attention effects had a maximum in parieto-occipital cortex (see Table S1 for coordinates) and was evident both before and after stimulus onset. Because the parieto-occipital sulcus itself has been tightly linked to posterior alpha oscillations [29, 30] and even cholinergic neuromodulation [26, 27], we had a closer look at this region and placed a spatial filter in the left and right bank of the parieto-occipital sulcus. This revealed a clear maximum in a cholinergic enhancement effect on spatial attention in the poststimulus phase in the classical alpha band (peak frequency of 10 Hz, see Figure S1, p < 0.005). Notably, despite the fact that some of these effects were clearly in the poststimulus phase, the observed attentional lateralization and its enhancement by the cholinergic agonist was largely independent of stimulus-evoked components (see also [4]), as indicated by the fact that subtraction of the latter from individual trials did not change these results (Figure S1).

### No Cholinergic Modulation of Gamma Attention Effects in Visual Cortex

**Figure 3:**
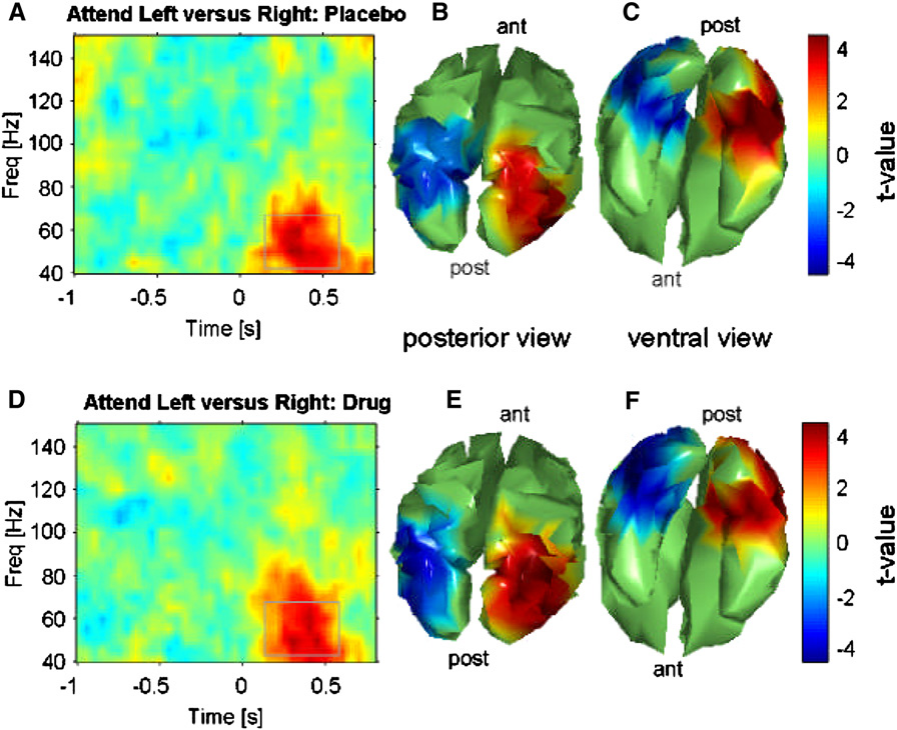
Spatial Attention and Gamma Oscillations (A) Time-frequency profile for symmetric hemispheric lateralization effects of Attention Left minus Attention Right for high frequency oscillations under placebo. (B and C) Topography of the high-frequency spatial attention effects under placebo for the time-frequency window marked in (A), shown in posterior view (B) or shown in ventral view (C), i.e., seen from below. Note that hot colors in the topographies indicate enhanced power contralateral to the attended hemifield, cold colors indicate reduced power ipsilateral to the attended hemifield. (D–F) Corresponding data now shown under physostigmine. Note the high reproducibility of the spatial attention effects on gamma, identical under drug/placebo. As a consequence there was no significant enhancement of gamma attention effects by the drug (the nonsignificant trend was actually for slightly stronger gamma attention effects under placebo). All values plotted are t values for the contrast of Attention Left minus Attention Right. Topographies are thresholded at p < 0.05, uncorrected, but for symmetric voxel pairs (see Experimental Procedures). See also Figure S2.

Consistent with previous reports [1–4], we found lateralized effects due to attended hemifield on gamma activity for visual cortex (p < 0.0001, uncorrected), extending into lateral occipital and ventral occipito-temporal cortex; see Figure 3. These gamma spatial attention effects emerged rapidly after stimulus onset and then endured for ∼500 ms. But note that these gamma attention effects were clearly *not* enhanced by physostigmine here, being highly reproducible in both the drug and placebo sessions (see Figure 3), with no significant difference (p > 0.2, was actually for slightly reduced gamma attentional effects under physostigmine). Likewise, stimulus-related visual gamma responses, due merely to onset of the visual gratings independent of attended hemifield, were also unaffected by physostigmine (see Figure S2). We note for completeness (and to show that gamma elsewhere could be affected) that there was a clear enhancement of a poststimulus-induced gamma-band response in frontal cortex (p < 0.01, uncorrected, see Figure S2). The impact of the drug on oscillations in early visual cortex was thus highly specific for the alpha/beta bands.

### Brain-Behavior Relations Induced by the Cholinergic Agonist

Finally we turned to possible relations between the neurophysiological effects and performance effects of our cholinergic intervention. We correlated the participant-by-participant drug effect on inverse efficiency scores (combining response speed and accuracy) to each of the neurophysiological effects described above (and as depicted in Figures 2, 3, S1, and S2) for the t-f windows shown. Note that these t-f windows had been selected independent of behavior, based either on attentional contrast in agreement with the literature or the difference between drug and placebo (Figures S1 and S2). The only significant brain-behavior relation observed was between drug-related performance speeding and the drug-induced poststimulus alpha spatial attention effects in the parieto-occipital sulcus (r = 0.65, p < 0.01). Although the effect in the extended time-frequency window in the lateral parts of parieto-occipital cortex as shown in Figures 2E and 2F were not significantly related to the behavioral effects, the poststimulus aspect (0–200 ms) was significant here, too (r = 0.52, p < 0.05). Given the limitation of this correlation to the poststimulus period, we further investigated whether this effect might by itself depend on any stimulus-evoked components but also checked on general effects of alpha/beta power irrespective of spatial attention. To this end we subtracted the stimulus-evoked field from the spectrograms (as in Figure S1) and computed a partial correlation analysis removing any general effects of alpha/beta power. Figure S3 shows the scatterplot for this analysis and reveals that the partial correlation for alpha lateralization in the parieto-occipital sulcus increased to r = 0.71 (p < 0.01) but decreased for the lateral aspects of parieto-occipital cortex (r = 0.41, p > 0.05). Thus, the key impact of the cholinergic agonist was upon alpha/beta oscillations modulated top-down by spatial attention in visual cortex. By contrast, gamma oscillations in visual cortex were unaffected.

## Discussion

**Figure 4:**
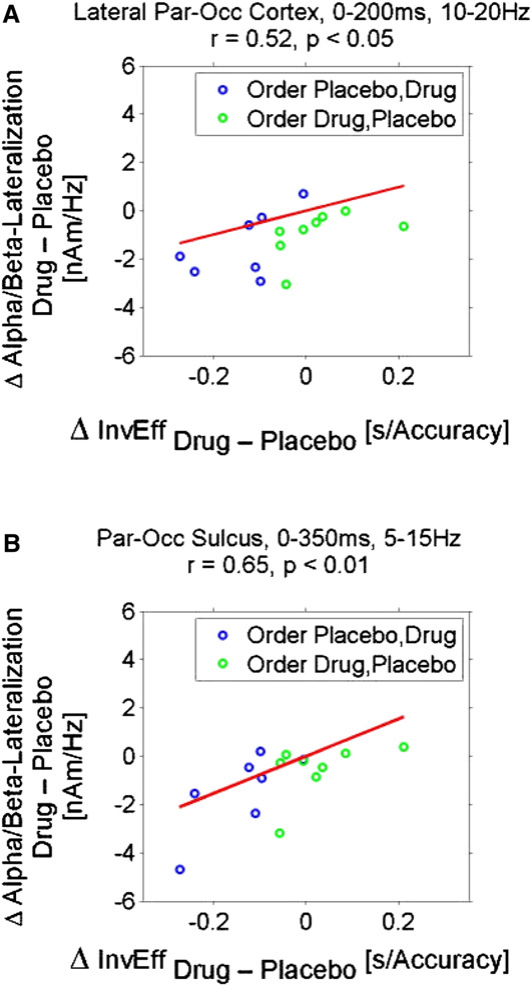
Brain-Behavior Relations Scatterplots with regression lines showing significant correlation of drug impact on poststimulus alpha/beta spatial attention effects with inverse efficiency scores for parieto-occipital cortex (see Figures 2E, 2F, and S1). (A) Correlation with the lateral parts of parieto-occipital cortex (Figure 2F, 10–20 Hz, 0–200 ms). (B) Correlation with an ROI in the parieto-occipital sulcus (Figure S1), a structure tightly linked with alpha oscillations at the t-f window where the drug effect is maximal there (5–15 Hz, 0–350 ms). Difference of attentional lateralization (Attention Left minus Attention Right) in power for right minus left hemispheres are shown on the *y* axis, differences of inverse efficiency is shown on the *x* axis. Each point gives difference scores for one participant, in blue the subjects where the drug session followed placebo and in green where drug preceded placebo. Negative values on the *x* and *y* axis indicate stronger effects in the expected direction (stronger hemispheric lateralization and faster processing for the physostigmine condition). Subjects for whom the drug was administered in the second session tend to have stronger effects. See also Figure S3.

Here we demonstrate via a causal intervention with a cholinergic agonist (physostigmine) that cholinergic neuromodulation augments the top-down impact of spatial attention on oscillations in human visual cortex, specifically for low-frequency alpha/beta bands. Previous studies show that cholinergic agonists enhance the hemodynamic BOLD response [10, 11] to attended stimuli in visual cortex or spike-rates recorded invasively [13] in primary visual cortex but the studies had not examined oscillatory phenomena. Although our results show the same pattern of spatial attention effects as a previous MEG study on spatial attention [4]—contralateral suppression (or/and ispilateral enhancement) of alpha/beta oscillations and contralateral enhancement of gamma oscillations—we show that a cholinergic enhancement via physostigmine boosts attentional alpha/beta effects in human visual cortex (Figure 2) but did not impact gamma effects in visual cortex (Figure 3; see also Figure S2). Moreover, the cholinergic impact on alpha/beta spatial attention effects were correlated to a drug-induced improvement in performance (Figure 4), such that strong attentional lateralization coincided with more efficient task processing, whereas any potential drug effect on visual gamma phenomena did not show such a correlation. Our alpha/beta findings provide a new line of evidence for the emerging view that low-frequency oscillations in visual cortex (and sensory cortex more generally) play a key role in gating sensory processing [6, 22, 31]. The specific relation to the drug-enhanced performance speeding here indicates that the cholinergic impact on attentional alpha/beta effects is not merely epiphenomenal.

In contrast to the impact on alpha/beta attention effects, the robustly observed gamma effects resulting from attended hemifield in visual cortex were *not* modified by the drug. This is a surprising outcome for theories [17–19] proposing that cholinergic neuromodulation impacts attentional selection by modulating gamma synchrony in particular. But those proposals were probably influenced by findings from hippocampus [14–16] or auditory cortex of anesthetized animals [24, 25] after cholinergic manipulations, not from recordings in visual cortex during an attention task with a cholinergic intervention. Moreover, the one invasive study to date [32] that examined cholinergic modulation of visual cortex while recording oscillations (albeit in anesthetized cats, without any attention task) found no immediate effect on the visually driven gamma response, analogous to our results (Figure S2) for awake humans in a cognitive task that allowed us to document spatial attention effects also (Figure 3). Note that we did, however, find an enhanced gamma-band response (after grating onset) in right frontal cortex (Figure S2), a brain structure that is intimately involved in control of attention [33] although it did not correlate with the performance speeding here. Likewise, in rats, frontal gamma oscillations may also depend on cholinergic activation [34].

Our findings suggest that cholinergic enhancement affects oscillatory activity in specific frequency bands, but differentially for distinct brain regions. This may relate to differential distribution of cholinergic receptors [35] and/or regional differences in circuitry, e.g., laminar activation patterns. One potential explanation for this arises from recent monkey studies [20, 21]. These highlight that gamma synchrony in visual cortex involves superficial (supragranular) feedforward layers, whereas alpha/beta synchrony involves predominantly the deeper (infragranular) feedback receiving layers. In the context of the present finding of cholinergic influence on alpha/beta, but not gamma, oscillations within human visual cortex, this raises the intriguing possibility of cholinergic enhancement primarily impacting feedback layers in the context of visual attention [35, 36]. Feedback influences are presumably key to top-down attentional influences. Although some proposals [37, 7] have emphasized enhanced bottom-up processing because of cholinergic modulation, other accounts propose cholinergic enhancement of attentional influences [8–13]. Our neurophysiological findings for human visual cortex document an example of the latter influence, yet, interestingly, we see this effect to extend well into the poststimulus period, suggesting a cholinergic impact on the interaction of bottom-up and top-down influences.

The drug used here, physostigmine, influences both nicotinic and muscarinic receptors [8], and it may be of interest to further distinguish the specific contributions of these in future work. Nevertheless, physostigmine has proven useful for studying the impact of the cholinergic system on neural processing in many previous studies [8–11] and is of particular interest as a drug applicable to humans. The importance of our results is that they provide the first evidence on how the cholinergic system modulates cortical oscillations, in the context of a visuospatial attention task, illustrating the power and potential of combining neuropharmacology with MEG [38, 39] and documenting the importance of low-frequency (alpha/beta) oscillations for visual attention.

## Experimental Procedures

### Participants

Sixteen healthy male volunteers (mean age 25.6 years, SD 5.7 years) participated after informed consent in accord with ethical clearance. Participants trained on the task and then performed two MEG sessions: one under drug, one with placebo in a double-blind crossover design.

### Task

Two visual gratings appeared, one in each hemifield centered at 8 degrees eccentricity (see Figure 1). Each trial started with a precue (central arrow pointing left or right for 500 ms) followed by a cue-target interval (length varied uniformly and unpredictably from 800 to 1200 ms), then onset of bilateral gratings for 500 ms. The task was to judge a tilt-offset for the grating in the cued hemifield (clockwise or counterclockwise relative to the diagonal), as indicated by pressing a right or left button with the corresponding index finger as quickly and accurately as possible. The actual tilt offset was titrated to yield ∼90% correct performance; see Supplemental Experimental Procedures for further details.

### Procedure

For the pharmacological MEG sessions, the responsible physician administered either the drug (0.01 mg physostigmine per kg bodyweight and infusion time and 0.2 mg glycopyrrolate as a peripheral antagonist; see Supplemental Information and [10, 11]) or the equivalent amounts of a saline solution for placebo via an intravenous line.

### Behavioral Data Analysis

We performed a regression analysis on the difference between drug and placebo in RT with drug/placebo session order as a covariate. The same analysis was performed for inverse-efficiency behavioral scores [36], which combine RT and accuracy as RT divided by proportion correct.

### MEG Recording and Analysis

MEG data were recorded continuously with a CTF Omega system at sampling rate of 600 Hz and analysis was primarily implemented with FieldTrip [40], unless stated. Procedures for recording, preprocessing, and artifact treatment followed previous work closely [1] as further described in Supplemental Experimental Procedures. For source reconstruction, we used a single-shell forward model [41] that was derived from the cortical sheets of each participant by a nonlinear warp of their brain to the MNI brain via SPM8 [42]. We used a beamforming approach [43, 44] to project the sensor data onto a (spatially) downsampled cortical grid representation. We then performed a time frequency analysis of the time courses on source level. For low-frequency bands (2.5–40 Hz), a wavelet analysis was computed and for the high-frequency bands, a multitaper analysis was computed.

### Analysis for Spatial Attention Effects

In order to minimize false positives, by design we implemented the following formal procedure to test here for symmetrically lateralized spatial attention effects. To test for the main effect of spatial attention, t tests were calculated for the difference between “Attend_Left” and “Attend_Right” for each grid point in the respective time-frequency windows. The symmetry constraint was operationalized by retaining only grid points that showed a significant difference (p < 0.05) in the above contrast (Attend_Left versus Attend_Right) *and* had a *corresponding grid point* for the reverse contrast (see Supplemental Experimental Procedures for further details). Subsequent tests for any drug modulation of attention effects also had the mirror symmetry constraint on paired t tests of attentional hemispheric lateralization.

### Correlation of Physiological Measures with Behavior

We related the drug impact on inverse efficiency to the drug impact on those MEG results of interest already reported to avoid a blind search through the entire brain-time-frequency matrix.

## Supplemental Information

Document S1. Supplemental Experimental Procedures, Figures S1–S3, and Table S1
