# Supplementary material for: Cholinergic Enhancement of Visual Attention and Neural Oscillations in the Human Brain
Source: Curr Biol. 2012 Mar 6;22(5):397–402. doi: 10.1016/j.cub.2012.01.022 (PMC3314945; doi:10.1016/j.cub.2012.01.022)
Supplement: Document S1. Supplemental Experimental Procedures, Figures S1–S3, and Table S1 [file mmc1.pdf]

Current Biology, Volume *22*

Supplemental Information

Cholinergic Enhancement of Visual Attention and Neural Oscillations in the Human Brain

Markus Bauer, Christian Kluge, Dominik Bach, David Bradbury, Hans Jochen Heinze, Raymond J. Dolan, and Jon Driver

Supplemental Inventory

Supplemental Figure S1 – Alpha/beta band in parieto-occipital cortex, related to Figure 2

Supplemental Figure S2 – Stimulus related gamma band activity, related to Figure 3
Supplemental Figure S3 – Partial correlation analysis, related to Figure 4

Supplemental Table 1
Supplemental Experimental Procedures
Supplemental References

Drug modulation of attention effect in parieto-occipital sulcus

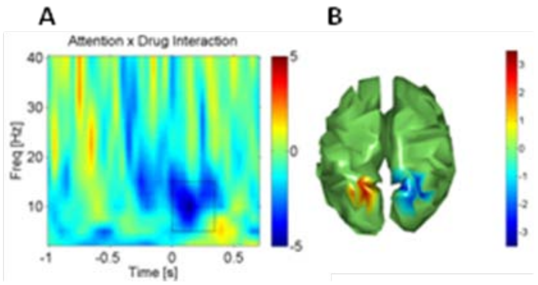

Attention: alpha/beta-band - Evoked field removed

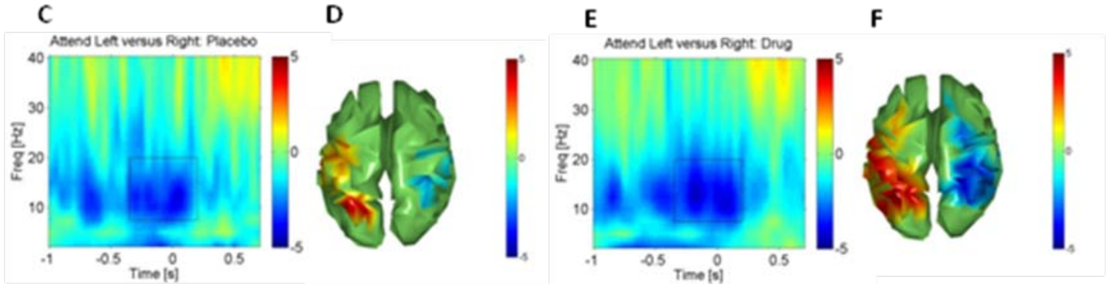

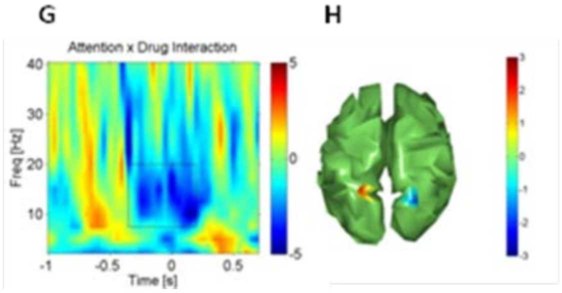

General alpha/beta event-related desynchronization **<u>independent of attentional state</u>**

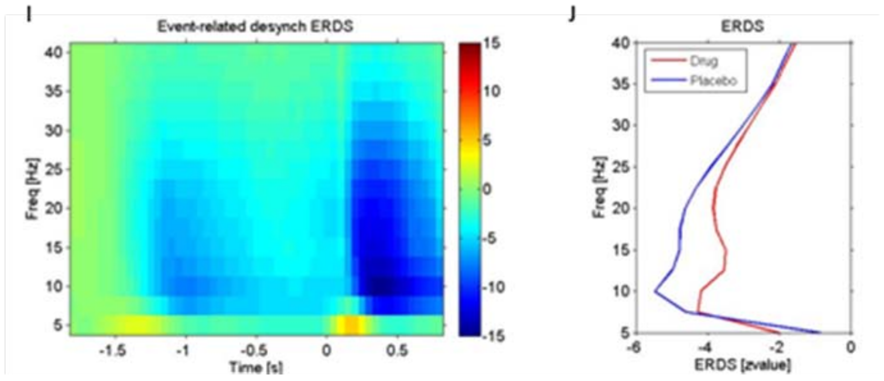

Figure S1 – Alpha/beta band in parieto-occipital cortex, **related to Figure 2**

(A-B): Cholinergic enhancement of attentional hemispheric lateralization as in Figure 3 E-F, but now for a ROI in the bank of the parieto-occipital sulcus: **(A)** The time-frequency (t-f) profile for

the drug related enhancement of the alpha/beta lateralization effect reveals a clear maximum in the post-stimulus phase, from approx. 0-300ms and a peak frequency of 10 Hz. **(B)** The topography shows the statistic of the effect in this window. Topography thresholded at $p<0.05$ with symmetry constraint.

(**C-H**) These panels are analogous to Figure 2 in the main manuscript but the evoked field was subtracted from individual trials to investigate whether the effect may have been driven by the frequency-representation of stimulus-locked evoked fields. The results confirm that the reported effects in the main manuscript are not driven by such evoked components. **(C)** The time-frequency (t-f) profile for the attention effect in the placebo condition. **(D)** The topography for the attention effect under placebo in the highlighted t-f window. **(E)** The time-frequency (t-f) profile for the attention effect in the physostigmine condition. **(F)** The topography for the attention effect under physostigmine in the highlighted t-f window. **(G)** The time-frequency (t-f) profile for the drug enhancement of the attention effect. **(H)** The topography for the drug enhancement of the attention effect in the highlighted t-f window (same as placebo). All plots show t-values and topographies thresholded at $p<0.05$, uncorrected but with symmetry constraint.

(I) Event related desynchronisation (locked to target onset) pooled over drug and placebo, independent of attentional state. **(J)** Frequency spectra in the time period from -350 to +200ms separately for drug and placebo. These figures show no clear separation for alpha- and beta-band.

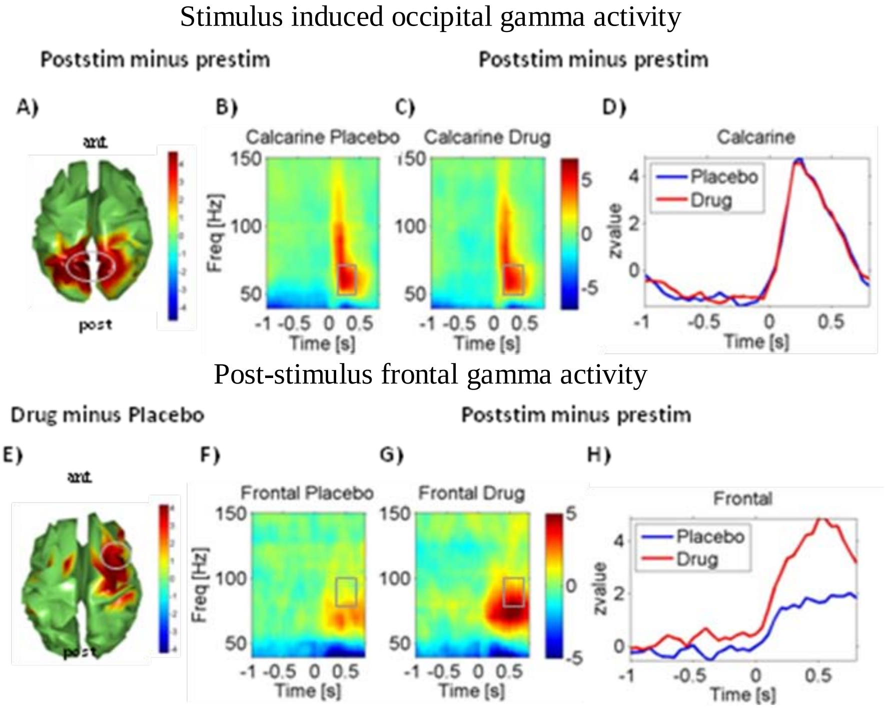

Figure S2 – Stimulus related gamma band activity, **related to Figure 3**

(A-D): Induced neural response to grating onset independent of attended side (i.e. pooled across attend left/right) for higher frequency bands. **(A)** Topography for stimulus-induced gamma increases regardless of drug (and of spatial attention), revealing robust posterior visual gamma responses in the period after visual target onset, corresponding to the window marked in the time-frequency profiles shown for early (peri-calcarine) visual cortex in **(B)** under placebo, and in **(C)** under physostigmine. Post=posterior aspect of brain, Ant=anterior. Note the very high reproducibility of the visually-induced gamma increase across placebo and drug conditions, Indeed there were no significant differences between visually-induced gamma under drug versus placebo, with the nonsigificant trend ($p>0.2$) actually being for slightly more gamma under placebo. **(D)** Shows timecourses of induced 50-70Hz gamma for these two conditions, again confirming the high reproducibility regardless of drug. Values plotted are z-values for post-vs pre-stimulus power. Topography thresholded at $p<0.01$.

(E-H): Induced gamma response as in (A), but now for the frontal areas that show an enhancement by the cholinergic agonist, in contrast to the unaffected gamma-band responses in visual cortex. **(E)** Topography for the statistical comparison between drug and placebo – showing an increased gamma-band response over (predominantly right) frontal cortex. **(F)** Time frequency profile of the response in area as marked in (E) under placebo, and in **(G)** under physostigmine. These are analogous to (B) and (C) but note here the clearly enhanced gamma-band response under physostigmine compared to placebo for right frontal cortex. This is also confirmed in timecourses **(H)** of induced 50-70Hz gamma for these two conditions. Values plotted are z-values for post-vs pre-stimulus power. Topography thresholded at $p<0.01$. Thus stimulus-induced gamma over right frontal cortex [45], a site implicated in the attentional control network, was influenced by physostigmine, in contrast with the unaffected gamma in visual cortex. This effect did not correlate significantly ($p > 0.1$) with behaviour or the attentional effects in visual cortex.

Partial correlation analysis on evoked subtracted data

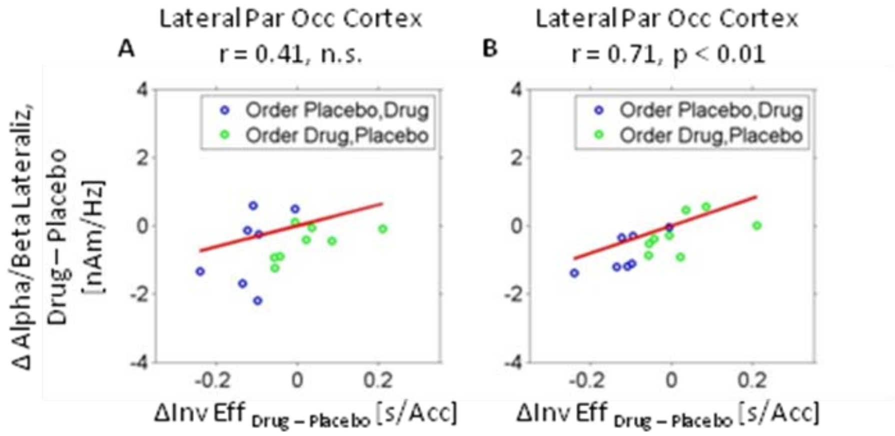

Figure S3 – Partial correlation analysis, **related to Figure 4**

These scatterplots are analogous to Figure 4 but depict the drug-modulation of the spatial attention effect after removal of drug effects on alpha-beta-power in the respective t-f-window (after the evoked field was removed). To remove the effect of the drug on alpha/beta-power a regression of the power values (collapsed over attentional conditions) on the drug effect on attentional difference was computed and the residual y-values were taken as the new attentional difference scores.

As in Figure 4, **(A)** shows the correlation with the lateral parts of parieto-occipital cortex (Fig.2F, 10-20Hz, 0-200ms). **(B)** shows the correlation with an ROI in the parieto-occipital sulcus (Supplemental figure S1), a structure tightly linked with alpha-oscillations at the t-f-window where the drug-effect is maximal there (5-15Hz, 0-350ms). Difference of attentional lateralization across drug and placebo condition (minus the drug effect on raw power) for right minus left hemispheres are shown on the y-axis, differences of inverse efficiency is shown on the x-axis. Each point gives difference scores for one participant, in blue the subjects where the drug session followed placebo and in green where drug preceded placebo. Negative values on the x- and y-axis indicate stronger effects in the expected direction (stronger hemispheric lateralization and faster processing for the physostigmine condition). Subjects for whom the drug was administered in the second session tend to have stronger effects.

Table S1: Centroid coordinates (MNI space)

	X (mm)	Y (mm)	Z (mm)
Beta Placebo centroid 1	34.06	-65.14	52.19
Beta Placebo centroid 2	33.21	-29.02	66.87
Beta Placebo centroid 3	41.68	-20.80	40.12
Beta Placebo centroid 4	61.29	-43.52	23.15
Beta Interaction lateral parieto occipital centroid 1	22.56	-64.16	43.16
Beta Interaction lateral parieto occipital centroid 2	28.41	-81.68	38.31
Beta Interaction lateral parieto occipital centroid 3	59.19	-52.45	-9.64
Alpha Interaction medial parieto-occipital centroid	19.43	-74.62	32.32
Gamma Placebo centroid 1	19.53	-82.80	-15.08
Gamma Placebo centroid 2	33.78	-33.09	-13.32
Gamma Placebo centroid 3	27.77	-43.58	67.63
Gamma Placebo centroid 4	31.80	-77.14	33.25
Gamma Placebo centroid 5	39.90	-66.38	-5.02

These centroids reflect the centers of gravity of local maxima of symmetrical attentional hemispheric lateralization – combined for left and right hemispheres for more robust estimates - and here the values are given for the right hemisphere.

Supplemental Experimental Procedures

Participants

Participants were recruited from an online data-base and gave informed consent prior to participation with the right to exit the study at any time. Exclusion criteria included: neurological disorders or family history of epilepsy; asthma or other strong allergy; cardiovascular disease; metabolic disease; glaucoma; influence of other drugs. Sixteen healthy male volunteers (mean age 25.6 years, s.d. 5.7 yrs) participated after informed consent in accord with ethical clearance. Females were not included as menstrual cycle may impact on drug sensitivity [35]. Participants trained on the task and then performed two MEG sessions, one under drug, one with placebo. These sessions were separated by at least two days, with order of drug or placebo counterbalanced across participants. Only the physician responsible for administering the agent/placebo knew whether it was placebo or drug, not the experimenter nor the participant.

Procedure and Drug administration

All participants underwent pre-training on the visual attention task (one or two sessions, until performance stabilized) with emphasis on maintaining central fixation and in order to determine the tilt-offsets yielding ~90% correct performance.

For the MEG sessions, participants were required not to drink alcohol the night before and no caffeine on the day of measurement. The responsible physician inserted the intravenous line before participants were brought to the magnetically shielded room (MSR). Participants then were sat comfortably in the MEG environment with peripheral electrodes (electrooculogram and electrocardiogram), head-localization coils and pulse-oxymeter attached. For drug sessions first 0.2mg Glycopyrrolate (a peripheral antidote to the cholinergic agonist Physostimgine that does not cross the blood-brain-barrier, see [10,11]) diluted in 5ml of saline was injected, while during

Placebo the same amount of pure saline was injected, via a 3-way port attached to the canule. The intravenous line was then attached to the pump (outside the MSR) and continuous infusion of either Physostigmine in a saline solution or else pure saline (Placebo) started at a rate titrated to individual bodyweight. Either 1 or 2mg Physostigmine was diluted in a 50 ml syringe filled with saline and the solution administered at a rate corresponding to 0.01 mg Physostigmine per kg bodyweight per hour (e.g. for a participant weighing 70 kg, 1ml Physostigmine was diluted in 50ml saline and infusion rate set to 35.7 ml solution per hour, corresponding to 0.7 mg Physostigmine per hour). Total infusion time was 70 min, starting 25 min prior to the experiment (to reach a more stable level of physostigmine plasma-levels, see [10,11]) then continuing for a further 45 min to limit the total dose given (see [46]). The experiment continued for another 15 min (half-time of Physostigmine plasma concentration is approximately 1-2 hours). In the 25 minutes prior to the experiment the seating position was optimised, the participant practised the task again and the head was localized. During the experiment heartbeat was continuously monitored via ECG and pulse-oxymetry. Placebo and drug sessions were separated by at least 2 days, in fully counterbalanced order.

Task

The task was to judge whether the grating on the attended side (as precued at trial start) was tilted clockwise or counterclockwise from diagonal. The grating in the currently unattended hemifield had an independent tilt (compared to the one in the attended hemifield) from diagonal but the global orientation of the gratings (top pointing to left or right) in the two hemifields was always opposite, to preclude any temptation of judging the grating presented in the irrelevant hemifield.

We used two cohorts of participants (n=8 each), each undergoing counterbalancing of drug/placebo order. In both cohorts the target tilt-offset during MEG sessions was initially chosen to yield ~90% correct performance as achieved during pre-training. In the first cohort we used a staircase

procedure during MEG, whereby < 75% correct performance over 24 trials would lead to an increase in the tilt-offset of 0.25 degrees, while >90% performance would lead to a corresponding reduction in tilt. This yielded actual mean tilt-offsets that did not differ between drug and placebo sessions (means 5.46 and 5.71 degrees for drug and placebo, respectively, s.d.s of 2.42 and 2.63 degrees). Since staircasing did not lead to significant drug/placebo differences in tilt-offset for the first cohort, for the second cohort this offset was kept constant, with its value again chosen to yield ~ 90% correct performance based on pre- training. MEG results were equivalent for the two cohorts, hence pooled.

<u>Behavioural data analysis</u>

We performed a regression analysis on the difference between drug and placebo in RT with drug/placebo session order as a covariate. Participants were faster in their second than first session (770.8 vs 828.3 ms); $t(15)=2.69$, $p<0.01$. They were faster under physostigmine than placebo (779.8 ms vs 819.2); $t(15)=-1.84$, $p<0.05$. The same outcomes were found if analyzing inverse-efficiency behavioral scores [36] instead, which combine RT and accuracy rate as RT divided by proportion correct ($t(15)=2.52$, $p<0.05$ for the drug effect, and $t(15)=3.6$, $p<0.01$ for the order effect).

<u>MEG recording and analysis</u>

<u>Recordings</u>

Data were recorded continuously from 274 axial gradiometers (1 of the SQUIDs from the sensor array over the head was defunct) plus 35 reference channels, using a CTF Omega whole head system at sampling rate of 600 Hz. Head position was measured via three coils, one placed at the

nasion and two attached to in-ear-tubes from the Etymotics system, see [3] at the beginning and end of each run.

Preprocessing

MEG data (3rd order axial gradiometer) were processed using FieldTrip http://www.fieldtrip.nl [40], being epoched and then checked for EOG-artefacts, muscle artefacts and amplitude-jumps using Fieldtrip partial artefact removal. The rejection threshold was set semi-automatically as described elsewhere [3]. Finally, whole trials were then visually inspected for any remaining artefacts, using Fieldtrip 'rejectvisual'. All artefact rejection was done blind to attention-condition (needed to be separate for drug condition as these were different sessions).

Forward-modelling

For source analysis, individual participants' T1 weighted MRI images were used to derive a mesh (8296 grid points) of the cortical surface and to calculate an individual forward model. Datasets were imported into SPM8 http://www.fil.ion.ucl.ac.uk/spm [42] for coregistration of MEG and MRI, segmentation and cortical mesh-calculation. We used the option of warping the canonical segmented MNI-brain onto the individual MRI to get a volume-conduction-model based on the approach proposed by [41]. This has been shown to provide comparable or sometimes even better source-analysis results than when individually segmented brains are used to calculate the volume-conductor model [47] and is a standard option in SPM8 [42]. It provides a homologous representation of the cortical sheet across participants allowing efficient grandaveraging and associated statistics.

The so derived head- and volume-conductor-models were then imported into FieldTrip (see http://www.fieldtrip.nl) and forward models were calculated with orientation fixed to the direction of the largest projected power (a standard option in fieldtrip 'sourceanalysis').

Source and frequency analysis

All main analyses were performed directly in source space (although we separately performed a sensor-level analysis with interpolated synthetic planar gradients, as in [3], that gave highly convergent results). The representation of the cortical sheet (as 8296 grid points) was downsampled (using 'reducepatch.m' in MATLAB), resulting in a representation of 413 grid points. This number only mildly exceeds the 274 sensors while being less than the 548 planar gradiometers (vertical and horizontal planar gradients) on which frequency analyses would otherwise have to be calculated [3]. For transformation into source-space, we used a time domain linear beamforming approach called linearly constrained minimum variance filtering [44]. In this approach a spatial filter (beamformer) is calculated from the covariance matrix of the sensor data and the leadfield according to the following formula:

$$\boldsymbol{w}(\boldsymbol{r}) = \left(\boldsymbol{h}(\boldsymbol{r})'(\boldsymbol{C}+\lambda\boldsymbol{I})^{-1}\left(\boldsymbol{h}(\boldsymbol{r})\right)^{-1}\boldsymbol{h}(\boldsymbol{r})'(\boldsymbol{C}+\lambda\boldsymbol{I})^{-1} \quad (1)$$

where **r** denotes the position-vector of the respective gridpoint, **h** is the leadfield matrix for a given point (orientation disregarded, but see below), **C** is the covariance matrix of the axial gradient sensor data, **I** is the identity matrix, λ is the regularization parameter and **w** is the resulting spatial filter (or beamformer) coefficient vector. In our analyses, lambda was set to half the mean of the diagonal of the cross-correlation matrix, a heuristic that yielded smooth sensor-topographies of the filter coefficients without signs of 'overfitting' in all participants. Instead of the inverse of the regularised covariance matrix, the Moore-Penrose pseudoinverse was taken, as standard in Fieldtrip. We calculated two sets of filters for each MEG dataset (i.e. each datafile), one for low-frequencies the other for high-frequencies (see below), irrespective of attentional condition or time-in-trial. This approach has been shown to give particularly robust spatial filters [48].

For *low-frequency analyses* (alpha/beta) the time-series of the (non-realigned axial gradiometer data) were baseline-corrected over the entire epoch then low-pass-filtered to 40 Hz with a sixth-order Butterworth filter and the covariance matrix computed across the entire trial. The nonrealigned axial gradiometer data were multiplied with these filter coefficients (i.e. linear projection), resulting in 413 time-series for each grid point (the orientation at each grid point was chosen along the dominant direction of the filter-coefficients for each dipolar orientation, again a standard option in the Fieldtrip 'sourceanalysis' routine). A wavelet analysis was run where a complex exponential of frequencies from 2.5 to 40 Hz in steps of 2.5 Hz was multiplied with a Hanning taper (rather than a Gaussian as in the classical Morlet wavelet), with length 3 cycles of the base frequency (e.g. 300 ms for 10 Hz) from -1s before gratings onset until 0.8s after grating onset. The use of a Hanning taper rather than the classical Gaussian enables shorter time-windows and reduces data losses at window edges otherwise caused by the longer window cycles needed for the Gaussian (the Hanning instead tapers to zero at edges).

For *high-frequency analyses* (gamma), covariance was calculated for bandpass-filtered data (40 – 150 Hz). Fixed time-windows were used with discrete prolate spherical slepian (DPSS) tapers to optimize smoothing in time and frequency domains and to suppress noise outside the time-frequency period of interest [49]. The window length was 200 ms for all frequencies (from 40 to 150 Hz in steps of 5 Hz) and we used 3 tapers (resulting in approx 10 Hz smoothing in the spectral domain) over the same time-window as for low-frequencies.

To obtain stimulus induced changes of oscillatory activity the spectra were baseline-corrected according to the following method: The baseline was calculated separately for each frequency and grid point at the time-window centered around 300ms prior to cue onset and then subtracted from the spectra of the peri-stimulus period. For each subject t-values were then obtained by dividing the

baseline-subtracted power values by the combined standard error of the mean of baseline and placebo. These t-values were then converted to z-values and averaged across subjects.

Removal of evoked fields:

To investigate whether some of the effects were driven by responses phase-locked to the stimulus ("evoked") we first calculated for each subject the frequency domain representation of the evoked field – by simply averaging the complex Fourier-coefficients (rather than the magnitude of these as resulting in the power-spectrum). These averaged complex Fourier spectra (for each time-, frequency-bin and grid point) were then subtracted from the single trial Fourier-spectra and then the power-spectrum was computed for each individual trial and then averaged for each subject. This approach is numerically equivalent to calculating the power spectrum of the (unfiltered) evoked field and subtracting this from individual trials power spectra but was simpler to accomplish in our analysis flow (see also [4] for a similar analysis).

Analysis of spatial attention effects

Main Effect of Attention (regardless of Drug)

To test for symmetrically lateralized spatial attention effects on visual cortex, we assessed effects that were present for Attend Left versus Right (and vice-versa) in corresponding regions of the right and left hemisphere. To this end we first calculated random effect paired t-tests on power-spectra for the contrast 'Attend_Left (L) minus Attend_Right (R)' according to the formula

$$t = \frac{mean_L - mean_R}{stdErrDiffMean_{L-R}} \quad (2)$$

where '$mean_L$' and '$mean_R$' refer to the average (across subjects) power-spectral-density-values for 'Attend_Left' and 'Attend_Right' conditions and '$stdErrDiffMean_{L-R}$' refers to the standard error of the difference of the means. After selecting time-frequency-windows where this difference (the main effect of spatial attention *pooled across drug and placebo conditions,* hence unbiased with

respect to any drug impact) was pronounced (in close agreement with the existing literature on spatial attention effects on oscillations, [1-6]), we then tested for the mirror symmetric modulation of oscillatory power by spatial attention. To this end grid-points were only retained if they showed a significant difference ($p < 0.05$) in the above contrast (equation 2) AND had a *corresponding grid point* on the other cortical hemisphere. Corresponding grid points were defined as those lying within a sphere of 17mm radius (close to the nearest-neighbour distance for the downsampled grids) at the mirror-symmetric position in the other hemisphere which showed a significant attention effect (according to equation 2) but of opposite sign to the first hemisphere. The mirror-symmetric position is defined as the position at the same y-, z-coordinates (MNI coordinate system: x-axis for left-right, y-axis for anterior-posterior and z-axis for ventral-dorsal direction), but with the opposite sign x-coordinate. Searching within a sphere was necessary since no exactly mirror-symmetrical grid-points arise in the downsampled representation of cortical gridpoints.

For the time-frequency images (as shown in figures 2 and 3 in the main paper) in order to combine effects from left and corresponding right hemisphere regions into one summary value, we performed the following calculation where $t_{RightHemisphere}$ and $t_{LeftHemisphere}$ refer to the result from equation 2 for the significant gridpoints from the right and left hemisphere, respectively:

$$t_{combined} = t_{RightHemisphere} - t_{LeftHemisphere} \qquad (3)$$

Statistical inference was then assessed by calculating the following test-statistic, averaging over time- (t), frequency- (f) and grid-points (s) of interest:

$$AttEff_{combined} = \sum_s^S \sum_t^T \sum_f^F ((AttL_{RightHem}(s,t,f) - AttR_{RightHem}(s,t,f)) - (AttL_{LeftHem}(s,t,f) - AttR_{LeftHem}(s,t,f)))/(F * T * S) \qquad (4)$$

where $AttL_{RightHem}(s,t,f)$ in equation 4 corresponds to the average power-spectral density of a right hemispheric grid point *s* at time-bin *t* and frequency bin *f* over trials where the left hemifield was

attended (and correspondingly $AttR_{LeftHem}(s,t,f)$ the average power-spectral-density of a left hemispheric grid point over trials where the right hemifield was attended.

This attentional lateralization index was then tested against zero with the following t-test:

$$t = \frac{Mean(AttEff_{combined}) - 0}{StdErrMean(AttEff_{combined})} \qquad (5)$$

$Mean(AttEff_{combined})$ denotes the average of the attentional lateralization index (equation 4) over subjects and $StdErrMean(AttEff_{combined})$ correspondingly the standard error of the mean of these values.

Drug modulation of attention effects

For assessing drug impacts on symmetric lateralized attention effects, we calculated the difference between attentional lateralization of power in Drug vs Placebo condition:

$$A_{DrugModulation} = [Att\_L - Att\_R]_{Drug} - [Att\_L - Att\_R]_{Placebo} \qquad (6)$$

and then assessed the significance of a deviation of this value (equation 6) again combined for the two hemispheres (in analogy to equation 4) against zero. This was performed in those regions-of-interest (time-frequency aspects of visual cortex) where we had already observed symmetric main effects of attention, regardless of the drug-manipulation (hence unbiased for the drug versus placebo contrast):

$$t_{DrugModulation} = \frac{Mean(A_{DrugModulation}) - 0}{StdErrMean(A_{DrugModulation})} \qquad (7)$$

t-values reported in the main text for drug impacts on attentional lateralization are the outcome of averaging power-spectral-densities in the same space-time-frequency-space region of interest as identified for the main-effects of attention (hence unbiased for potential drug-placebo difference) and then performing the new test for significance of the drug impact (equation 7).

For the effect shown in the parieto-occipital sulcus shown in Supplemental figure 1, a region of interest (ROI) was located in the left and right bank of the parieto-occipital sulcus and spatial

attention effects for this were then calculated in the same way as described above. We investigated this region more closely because of its known relevance as the key generator of alpha-oscillations in visual cortex and because previous studies have shown that cholinergic neuromodulation may impact on alpha-beta-oscillations there [26,27].

Estimation of centroids of attentional hemispheric lateralization results

In order to give meaningful coordinates of those brain regions significantly modulated by attention (or attention being significantly modulated by the drug) we developed a procedure as described below. This seemed necessary given the spatially extended nature of the observed attentional modulations – where mean coordinates of all significant grid points would not necessarily be very meaningful given the 3D folded nature of the brain.

We searched across the entire cortical representation for local maxima in the statistics for attentional differences. These local maxima were defined as those grid points that were only surrounded by neighbouring grid-points with lower absolute t-values. Then we calculated the (weighted) mean coordinates of all significantly modulated grid points within a sphere centered around the respective local maximum – weighted by their attentional modulation strength (t-value). To give more robust estimates of these centroids we collapsed the statistic across both hemispheres and report here the coordinates for locations in the right hemisphere.

Brain-behaviour relations

We calculated for each participant (and session, i.e. drug or placebo) their average for the same time-frequency windows as marked in the Results figures (fig. 2-3) of the main paper. We then correlated these participant-by-participant physiological effects, due to the causal drug intervention,

with the corresponding inverse-efficiency scores (as well as reaction time, not reported here), using Pearson correlation coefficients.

We further tested the specificity of these correlations by 1) repeating the analysis on evoked-field removed data and 2) partializing the drug effect on alpha/beta-power out from the attentional lateralizations. The results of this analysis are shown in Supplemental Figure S3.

Supplemental References

45. Apparsundaram, S., Martinez, V., Parikh, V., Kozak, R., Sarter, M. (2005) Increased capacity and density of choline transporters situated in synaptic membranes of the right medial prefrontal cortex of attentional task-performing rats. J. Neurosci. *25*, 3851-6

46. Bentley, P., Driver, J., Dolan, R.J. (2008) Cholinesterase inhibition modulates visual and attentional brain responses in Alzheimer's disease and health. Brain. *131*, 409-24.

47. Henson, R.N. , Mattout, J., Phillips, C., Friston, K.J. (2009) Selecting forward models for MEG source-reconstruction using model-evidence. Neuroimage *46*, 168-76.

48. Litvak, V., Eusebio, A., Jha, A., Oostenveld, R., Barnes, G.R., Penny, W.D., Zrinzo, L, Hariz, M.I., Limousin, P., Friston, K.J., Brown, P. (2010) Optimized beamforming for simultaneous MEG and intracranial local field potential recordings in deep brain stimulation patients. Neuroimage. *50*, 1578-88.

49. Mitra, P.P., Pesaran, B. (1999) Analysis of dynamic brain imaging data. J Biophys *76*, 691-708.
